# In Search for Boundary Conditions of Reconsolidation: A Failure of Fear Memory Interference

**DOI:** 10.3389/fnbeh.2017.00065

**Published:** 2017-04-19

**Authors:** Natalie Schroyens, Tom Beckers, Merel Kindt

**Affiliations:** ^1^Department of Psychology, KU LeuvenLeuven, Belgium; ^2^Department of Clinical Psychology, University of AmsterdamAmsterdam, Netherlands

**Keywords:** fear memory, destabilization, contextual novelty, reconsolidation, boundary conditions

## Abstract

The presentation of a fear memory cue can result in mere memory retrieval, destabilization of the reactivated memory trace, or the formation of an extinction memory. The interaction between the degree of novelty during reactivation and previous learning conditions is thought to determine the outcome of a reactivation session. This study aimed to evaluate whether contextual novelty can prevent cue-induced destabilization and disruption of a fear memory acquired by non-asymptotic learning. To this end, fear memory was reactivated in a novel context or in the original context of learning, and fear memory reactivation was followed by the administration of propranolol, an amnestic drug. Remarkably, fear memory was not impaired by post-reactivation propranolol administration or extinction training under the usual conditions used in our lab, irrespective of the reactivation context. These unexpected findings are discussed in the light of our current experimental parameters and alleged boundary conditions on memory destabilization.

## Introduction

Under specific conditions, presentation of a fear memory cue can result in destabilization of a previously consolidated memory. Afterwards, restabilization, a process often referred to as “reconsolidation,” should take place in order to ensure persistence of the memory trace. The (re)discovery of reconsolidation as a protein-synthesis dependent process drew attention to the malleability of memory and raised the possibility of interfering with existing memories during this temporary window of lability (Misanin et al., 1968; Nader et al., 2000). Such a manipulation would be of particular interest in targeting maladaptive fear memories in, for example, patients with phobias, panic disorder, or post-traumatic stress disorder (Beckers and Kindt, 2017). Indeed, overwhelming evidence in a wide variety of protocols and species has shown that neurobiological manipulations during or shortly after memory reactivation can change subsequent memory performance, possibly by interfering with reconsolidation (Finnie and Nader, 2012). In particular, there is ample evidence that post-reactivation administration of propranolol, a noradrenergic beta-blocker that indirectly targets protein synthesis, reduces the later expression of a fear memory in humans (Kindt et al., 2009; Soeter and Kindt, 2010, 2011, 2012a,b, 2015a,b; Sevenster et al., 2012, 2013, 2014).

However, destabilization does not appear to be a universal property of memory. Instead, presentation of a memory cue can also result in mere retrieval or extinction (Sevenster et al., 2012, 2013, 2014; Merlo et al., 2014). The outcome of a memory reactivation procedure depends on the interaction between the initial learning experience and reactivation conditions (Sevenster et al., 2013; Alfei et al., 2015). The window that serves the opportunity of targeting a memory is thus restricted, and the success of amnestic agents depends on subtle differences in the memory reactivation procedure. Despite the huge amount of studies that report successful amnestic effects, there have been recent reports of failures to induce amnesia by administering propranolol after fear memory reactivation (Bos et al., 2014; Thome et al., 2016). These conflicting findings have mainly been attributed to the presence of boundary conditions or methodological differences across studies, which will be discussed in more detail in the discussion. For the prospect of reconsolidation-based treatments, it is of crucial importance to reveal the conditions under which memories can become destabilized, and thus subject to change (Beckers and Kindt, 2017).

One important prerequisite for the induction of reconsolidation may be an optimal degree of match-mismatch between what has been learned and what is presented during reactivation (Finnie and Nader, 2012). Since one possible function of reconsolidation is to update existing memories, the presentation of new information during memory reactivation could be vital for triggering this process (Lee, 2010). As such, a prediction error, or a mismatch between actual and expected events that promotes further learning, may be required to trigger memory destabilization and subsequent reconsolidation (Pedreira et al., 2004; Morris et al., 2006; Sevenster et al., 2013). In the lab, this is typically achieved by re-exposure to the original fear-conditioned stimulus (CS), without administration of the anticipated unconditioned stimulus (US). At the same time, there should also be a certain amount of overlap (i.e., match) between learning and reactivation conditions. Especially in case of non-asymptotic learning, reactivation conditions should be sufficiently similar to the initial learning experience in order to induce reconsolidation-dependent memory updating. A reactivation session that is too different from original learning conditions might not cause memory destabilization, but initiate the formation of a new memory trace (Hupbach et al., 2008). Accordingly, environmental changes may produce a switch from updating (of an existing memory) to encoding (of a new memory trace).

The underlying neurological basis for the occurrence of memory updating vs. encoding can be located in the hippocampus and its role as a comparator between past and present experiences. When incoming contextual information does not sufficiently match a previously stored memory, “pattern separation” occurs and hippocampal dynamics shift toward an encoding state rather than a retrieval state (Hasselmo et al., 1996; Meeter et al., 2004; Lisman and Grace, 2005; Kumaran and Maguire, 2006). Therefore, the presentation of a reminder cue in a new environment may initiate the formation of a new memory trace (i.e., such as in fear extinction), as opposed to the updating of an existing memory (i.e., reconsolidation).

Indeed, several animal and human studies support the idea that the presence of novel contextual information during memory reactivation prevents reconsolidation from occurring. One of the early animal studies on reconsolidation showed that disruption of a conditioned fear memory by an electroconvulsive shock did not occur when the conditioned stimulus (CS) was presented alone during the reactivation session. Instead, the CS had to be presented within its training environment in order to successfully interfere with the original memory (DeVietti and Holliday, 1972). Another study in rats revealed that reactivation of a passive avoidance memory in the training context was more effective than reactivation in a new context (Rodriguez, 2000). Also in snails, it was shown that memory is only reactivated during re-exposure to the training context—but not a novel context—1 day after operant conditioning (Parvez et al., 2005). Interestingly, a human study by Hupbach et al. (2008) reported that updating of an episodic memory only occurred during re-exposure in the same context as the original learning, whereas memory reactivation in a novel context resulted in the creation of a new memory trace. These latter results imply that the spatial context during memory reactivation is a crucial factor in determining whether reconsolidation (i.e., interference with the original memory) or extinction (i.e., creation of a new memory trace) is induced. In line with this analysis, studies on fear memory extinction indeed show that extinction learning proceeds faster during cue exposure in a novel context, compared to cue exposure in the original training context (Vansteenwegen et al., 2005), suggesting that new learning is more rapidly initiated in a novel context. Contextual novelty during reactivation might thereby impose boundaries on the induction of memory destabilization and reconsolidation.

It should be noted that the previously discussed findings remain controversial, as many animal studies have reported successful reconsolidation during fear memory reactivation in a novel context (Nader et al., 2000; Dȩbiec and Ledoux, 2004; Tronson and Taylor, 2007). There is even evidence for an opposite effect of a novel reactivation context on reconsolidation. Two animal studies indicated that novel contextual information during reactivation was necessary to induce reconsolidation of a strong auditory fear memory and a strong or old object memory (Winters et al., 2009; Jarome et al., 2015, respectively). While the role of context in *episodic* memory reactivation seems to be clear (Hasselmo et al., 1996; Meeter et al., 2004; Lisman and Grace, 2005; Kumaran and Maguire, 2006; Hupbach et al., 2008), these latter results imply that the effect of context changes during reactivation is more variable in case of *fear* memories, as contextual novelty might under certain conditions facilitate rather than hamper the induction of fear memory reconsolidation.

To conclude, there is a vast amount of research suggesting that context plays a role in accessing the memory trace of a learned experience. Depending on the learning history, new contextual information can either *prevent* or *boost* the induction of reconsolidation by the presentation of a memory cue. For memories acquired by non-asymptotic learning, reactivation in a novel context might impose too much novelty and result in new learning. Strong memories, on the other hand, may require more novelty detection for destabilization to be induced, and a context change may therefore be necessary for their reconsolidation. A relevant question, which has remained unaddressed, is whether a novel context can act as a boundary condition on reconsolidation of human fear memories acquired through non-asymptotic fear conditioning. The current study adopted a 3-day differential fear-conditioning paradigm including post-reactivation administration of propranolol HCl, but this time we utilized different meaningful backgrounds on which the conditioned stimuli were presented as opposed to the usual black background in our previous studies. We hypothesized that introducing novel contextual information during reactivation following non-asymptotic learning would prevent destabilization of a fear memory that was previously shown to be sensitive to destabilization when reactivated in the original context. Thus, we expected that emotional expression of the fear memory, as measured by the eyeblink startle reflex, would be disrupted by propranolol only if the memory cue was presented in the training context during the reactivation session, but not when it was presented in a new context.

## Materials and methods

### Participants

Forty healthy individuals (30 females) ranging from 18 to 27 years old (*M* = 21.1, *SD* = 2.1) participated in the study. One participant was excluded due to a technical failure during fear conditioning. For two participants (from groups AAA and ABA), the program blocked during extinction trial 9 or 8, respectively. Three participants did not receive any shocks during reinstatement (two from group ABA and one from group AAA). Therefore, reinstatement test data from these three participants were excluded. All participants reported to be free from any condition contraindicative to the administration of electrical shocks or propranolol HCl. Participants were excluded if their blood pressure was lower than 90/60 mmHg or if heart rate was under 50 bpm, as measured at the beginning of session 1 and 2. Finally, participants with a score ≥26 on the Anxiety Sensitivity Index (ASI) were excluded as they might experience difficulties with any temporary symptoms induced by propranolol HCl. Participants were pseudo-randomly assigned to one of the two groups (AAA or ABA), with the restriction that groups were matched on their Fear of Spiders Questionnaire (FSQ) and the Trait Anxiety Inventory (STAI-T) scores. All participants received either partial course credits or 50 euros as compensation. All subjects gave written informed consent in accordance with the Declaration of Helsinki. The protocol was approved by the local ethics committee at the University of Amsterdam.

### Apparatus and materials

#### Stimuli

The conditioned stimuli (CSs) consisted of pictures of a spider, a gun, and a cup (Supplementary Material, Figure 1, adapted from IAPS: nr 1,201, 6,210, 7,009, respectively). The two fear-relevant stimuli (i.e., spider and gun; CS1 and CS2) served as CSs^+^, while the fear-irrelevant stimulus (i.e., cup; CS3) was used as the CS^−^. The CSs^+^ were repeatedly followed by an electrical stimulus to the wrist (2 ms, 80% reinforcement: 1st trial unreinforced), whereas the CS^−^ was not. The picture used as CS1 (spider or gun) was counterbalanced. Each stimulus was presented for 8 s. The use of 2 CSs^+^ allowed selective reactivation of one of two fear associations. Since propranolol has been shown to selectively interfere with fear memory for the reactivated CS^+^ (here CS1), the non-reactivated CS2 can be used as a within-subject control stimulus. The fear-irrelevant control cue was employed to test for successful fear acquisition, and to verify whether our propranolol manipulation was capable of neutralizing fear responding (Soeter and Kindt, 2011). The startle probe, a 40-ms duration noise burst (104 dB, rise/fall time shorter than 1 ms) was delivered binaurally through headphones (HD 25-1 II, Sennheiser) 7 s after CS onset. Inter-trial intervals (ITI) varied from 15 to 25 s with an average of 20 s. The order of stimulus presentation was fully randomized within blocks (i.e., CS1, CS2, CS3, NA: noise alone), except for each first test trial on day 3 (at the beginning and after reinstatement), which was fixed (and counterbalanced). The unconditioned stimulus (US) consisted of an electrical shock administered to the wrist of the non-preferred hand 7.5 s after CS^+^ onset. Intensity of the shock was determined individually as “clearly unpleasant but not painful” by a gradual work-up procedure. The shock level ranged from 1 to 62 mA (*M* = 22.7; *SD* = 14.2). Delivery of the shocks was controlled by a Digitimer DS7A constant current stimulator (Hertfordshire, UK) via a pair of Ag electrodes of 20 by 25 mm with an inter-electrode distance of 45 mm. A conductive gel (Signa, Parker) was applied on the electrodes.

**Figure 1:**
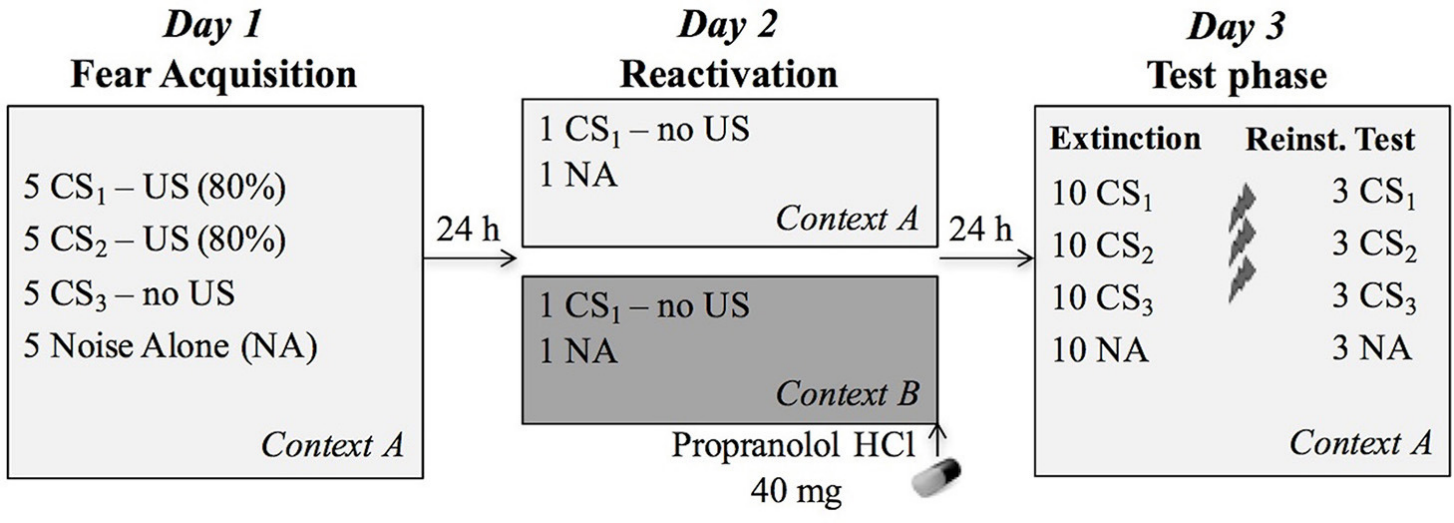
**Overview of the experimental protocol**. During fear acquisition on day 1, the CS1 (reactivated conditioned stimulus) and CS2 (non-reactivated conditioned stimulus) were followed by the US (unconditioned stimulus, electric shock) on four out of five presentations, while the CS3 (control stimulus) was never followed by a shock. During reactivation, participants received 40 mg propranolol HCl after a non-reinforced presentation of CS1 in the training context A (*n* = 20) or a new context B (*n* = 19). The next day, all CSs were presented again in the learning context A to assess differential fear memory retention, followed by an extinction session, reinstatement (3 shocks) and a reinstatement test.

#### Context manipulation

The context was manipulated by changing the background picture on which the CSs were presented. Pictures of a living room and a garden, occupying the entire computer screen, served as two different contexts (Supplementary Material, Figure 1). The context picture was continuously presented (incl. during habituation and noise alone trials). The picture used as acquisition context A was counterbalanced. Participants in group AAA were shown context A on each day, whereas participants in group ABA were shown context A on day 1 (acquisition) and 3 (extinction and reinstatement), and context B on day 2 (reactivation).

#### Fear-potentiated startle

Potentiation of the eyeblink startle reflex served as an index of conditioned fear responding and was measured through electromyography (EMG) of the right orbicularis oculi muscle in response to the startle probe that was administered during each CS presentation and during ITI (i.e., NA trials). A pair of Ag/AgCl electrodes (6 mm, BME-175, BIOMED) was filled with electrolyte gel (Signa, Parker) and positioned ~1 cm under the pupil in forward gaze and 1 cm below the lateral canthus. A ground reference was placed on the forehead (Blumenthal et al., 2005). EMG electrodes were connected to a custom-made bipolar EMG amplifier with an input resistance of 1G Ω and a bandwidth of 5–1,000 Hz (6 dB/oct). Both the raw and 50 Hz notch-filtered EMG were sampled, the filtered signal was used for data analysis. Peak amplitudes were identified during the 50–150 ms interval following probe onset. The software program Vsrrp98 was used for EMG data acquisition and reduction. The EMG channels were sampled at 1000 S/s (National Instruments NI-USB6210).

#### Online US expectancy measures

Participants were instructed to rate their shock expectancy during the first 5 s of each CS presentation by using a continuous rating scale, ranging from −5 (*certainly no electric stimulus*) to 0 (*uncertain*) to +5 (*certainly an electric stimulus*). The scale was present on the bottom of the computer screen during the entire experiment, but participants could only indicate their response during the 5-s interval after stimulus onset by shifting the cursor on the scale and pushing the left mouse button. Expectancy scores were transformed to a scale ranging from 0 (*certainly no electric stimulus*) to 100 (*certainly an electric stimulus*).

#### Blood pressure and heart rate

Blood pressure (BP) and heart rate (HR) were measured at the start of each session and at the end of session two, using an electronic sphygmomanometer (OMRON M4-I, Healthcare Europe BV, Hoofddorp, The Netherlands) with a cuff applied around the left upper arm. At each time point, the measurement was performed three times and the average BP and HR were calculated.

#### Pharmacological treatment

Propranolol HCl (40 mg) was obtained from the pharmacy (Huygens Apotheek, Voorburg, the Netherlands).

### Experimental procedure

In order to assess whether memory reactivation in a novel context affects propranolol's capacity to disrupt a 24-h old fear memory, we adopted a differential fear-conditioning paradigm that was previously developed in our lab. The protocol included testing over three different phases separated by 24 h (Figure 1). Each session started with a 1-min acclimation period consisting of 70 dB broadband noise, followed by 10 startle habituation trials in order to stabilize baseline startle responding. The 70-dB noise continued during the entire experiment in order to avoid distraction by surrounding sounds. The context (i.e., background image) was also presented throughout the whole experiment, including the acclimation phase.

#### Fear acquisition in context A (day 1)

After obtaining written informed consent and administration of the medical screening, the STAI, ASI, and FSQ were administered. If the participant did not meet any of the exclusion criteria, EMG and shock electrodes were attached, and the shock level was determined. The participants were instructed that two of the pictures would “most of the time” be followed by a shock, while the third picture would “never” be followed by a shock. In addition, they were instructed that they should learn to predict which pictures would (not) be followed by a shock, and to rate their shock expectancies during each picture presentation. Afterwards, they were presented with a series of pictures (conditioned stimuli, CSs). The fear-relevant pictures (i.e., spider and gun) were repeatedly followed by an electric shock (US, 80% reinforcement: 1st trial was unreinforced), whereas the picture of the cup was never followed by a shock. The use of two CSs^+^ allowed selective reactivation of one of the CSs^+^ (i.e., CS1) on day 2. Therefore, the non-reactivated CS2 could be used as a within-subject control for the reactivated CS1. All CSs were shown 5 times and presented within a specific context (i.e., background image of a living room or garden). It bears mentioning that the presence of such a background image differs from previous propranolol studies in our lab, as the IAPS pictures were usually presented on a black screen. Only one of those previous studies did apply simple background colors to the pictures in order to assess renewal of fear due to a context change (Soeter and Kindt, 2012a). After finishing the experimental task, contingency awareness was assessed by asking the participant which of the pictures were followed by a shock most of the time. They were also instructed to remember what they had learned about the stimuli. After detachment of the electrodes, subjects filled in the STAI-S and rated US unpleasantness. At the end of the session, they were instructed to (1) refrain from sporting and drinking caffeine or alcohol 12 h prior to session 2, and (2) refrain from eating or drinking (except water), smoking and using chewing gum 2 h prior to session 2. These instructions were used in previous propranolol studies in our lab to acquire unbiased saliva samples, and we adopted them in order to keep propranolol HCl absorption standardized between subjects.

#### Memory reactivation in context A or B (day 2)

Twenty-four hours later, subjects were selectively re-exposed to an unreinforced conditioned stimulus (CS1, without US), followed by the administration of an oral dose of 40 mg propranolol HCl. Afterwards, participants stayed in the lab for 90 min, during which they were offered magazines to read and were not allowed to do anything else. The memory reactivation session took place in either context A (i.e., acquisition context) or in a novel context B (i.e., group AAA or ABA, respectively). At the beginning and end of the session, participants filled in the STAI-S and BP and HR were measured.

#### Extinction and reinstatement test in context A (day 3)

During the last day of the experiment, participants first filled in the STAI-S and BP and HR were measured again. Afterwards, each stimulus was presented 10 times without reinforcement. Differential fear responding on the first CS1, CS2, and CS3 trial was used to assess retention of the fear memory. If propranolol successfully (and specifically) disrupted the memory, fear responding to the reactivated CS1 should be abolished (i.e., equal to CS3), while fear responding to the non-reactivated CS2 should remain relatively high. The subsequent unreinforced presentations constituted an extinction procedure. Finally, three unsignaled USs were administered, followed by reinstatement testing (3 trials of each stimulus). Day 3 took place in the acquisition context (i.e., context A). At the end of the experiment, participants filled in the STAI-S, rated unpleasantness of the US and the startle probes, and filled in a short self-made questionnaire about the instructions that were given by the experimenter on each day (clarity, trustfulness, believability on day 1, 2, and 3). Shock and startle probe evaluation questionnaires included four questions: (1) how unpleasant was the shock/sound to you? (2) how intense was the shock/sound? (3) to what extent did the shock/sound startle you? (4) how hard was it for you to tolerate the shock/sound?

### Statistical analysis

Startle data were standardized into z-scores in order to reduce between-subjects variability. Z-scores were calculated based on the average of all startle responses over all phases within subject, excluding habituation trials. Missing data points were excluded from the analyses and outliers (*z* > 3) were replaced by the linear trend at point for the specific stimulus within the relevant phase. Startle responses and US expectancy ratings were analyzed by means of mixed factorial repeated-measures analyses of variance (ANOVAs) with between-subjects factor group (AAA vs. ABA) and within-subject factors stimulus (CS1, CS2, and CS3) and trial (first vs. last trial of each phase). Simple contrasts were used to follow-up significant effects. STAI-T, STAI-S, ASI, FSQ, and US-intensity scores were subjected to independent-samples *T-*tests in order to check whether there were any differences between the groups. In order to assess propranolol effects on heart rate and blood pressure, mixed ANOVAs with between-subjects factor group and within-subject factor time (pre vs. 90 min post propranolol administration) were performed. The Greenhouse-Geisser correction was used when the assumption of sphericity was violated. Criterion for significance was set at 0.05 and partial eta squared (η_*p*_^2^) was used as effect size. All analyses were carried out using SPSS Statistics.

## Results

### Questionnaires

The groups did not differ in trait anxiety [*t*_(37)_ = 1.60; *p* = 0.118], state anxiety as assessed at day 1 before fear conditioning [*t*_(37)_ = 0.89; *p* = 0.380], anxiety sensitivity [*t*_(37)_ = 0.50; *p* = 0.623], reported spider fear [*t*_(37)_ = 0.10; *p* = 0.920], shock intensity [*t*_(37)_ = 0.76; *p* = 0.454], or rated shock unpleasantness [*t*_(37)_ = 0.86; *p* = 0.392; Tables 1, 2]. Subjective unpleasantness of the shock significantly decreased from day 1 to day 3 (i.e., the shock was rated less unpleasant on day 3) [main effect of day; *F*_(1, 34)_ = 21.59; *p* < 0.001; η_*p*_^2^ = 0.39], which indicates that some habituation to the shock took place (Table 2, first row). State anxiety significantly decreased from the beginning to the end of day 2 [main effect of time on day 2; *F*_(1, 35)_ = 11.65; *p* = 0.002; η_*p*_^2^ = 0.25; Table 3]. On day 1 and 3, state anxiety increased from the beginning to the end of the session [*F*_(1, 35)_ = 15.76; *p* < 0.001; η_*p*_^2^ = 0.31 and *F*_(1, 35)_ = 6.30; *p* = 0.017; η_*p*_^2^ = 0.15, respectively].

**Table 1:** **Participant characteristics (***N*** = 39)**.

**Participant characteristics**	**Group**			
	**AAA**		**ABA**	
	***M***	***SD***	***M***	***SD***
Age	20.9	2.4	21.4	1.8
Trait anxiety	32.4	6.1	35.7	6.9
Spider fear	36.3	23.1	36.9	19.5
Anxiety sensitivity	10.0	7.8	11.0	4.2
US intensity (mA)	21.1	14.1	24.6	15.0
*N*_female/total_	16/20		14/19	

**Table 2:** **Mean evaluation scores (SD) for the US and startle probe by the participants**.

	**Group**			
	**AAA**		**ABA**	
	**Day 1**	**Day 3**	**Day 1**	**Day 3**
**US EVALUATION**				
(Un)pleasantness [−5 (unpleasant) − 5 (pleasant)]	−3.7 (0.9)	−2.9 (1.2)	−3.4 (0.7)	−2.2 (1.3)
Intensity [1 (soft) − 5 (unbearable)]	3.2 (0.6)	2.7 (0.9)	3.1 (0.5)	2.5 (1.1)
Startlingness [1 (none) − 5 (very strong)]	4.0 (0.8)	3.7 (1.0)	3.8 (0.6)	3.5 (1.4)
Demanding effort to tolerate [1 (none) − 5 (a lot)]	3.2 (0.9)	2.7 (1.0)	3.1 (0.6)	2.4 (1.2)
**STARTLE PROBE EVALUATION**				
(Un)pleasantness [−5 (unpleasant) − 5 (pleasant)]		−3.3 (1.7)		−2.9 (1.8)
Intensity [1 (soft) − 5 (unbearable)]		3.0 (1.3)		2.9 (1.4)
Startlingness [1 (none) − 5 (very strong)]		3.8 (1.5)		3.4 (1.6)
Demanding effort to tolerate [1 (none) − 5 (a lot)]		2.8 (1.3)		2.8 (1.4)

*US, Unconditioned Stimulus (i.e., electric shock)*.

**Table 3:** **Blood pressure, heart rate, and state anxiety before and after propranolol intake**.

**Measure**	**Group**				**Results mixed ANOVAs**	
	**AAA**		**ABA**		**Time × Group**	**Time**
	**Pre**	**Post**	**Pre**	**Post**		
Systolic BP	112.8 (36.3)	105.2 (11.5)	111.8 (8.0)	103.5 (8.8)	*p =* 0.553	*p* < 0.001
Diastolic BP	75.2 (24.5)	72.4 (10.7)	74.0 (5.4)	71.0 (7.1)	*p =* 0.355	*p* = 0.005
Heart rate	73.0 (24.2)	54.1 (7.3)	71.5 (12.0)	51.2 (6.2)	*p =* 0.317	*p* < 0.001
State anxiety	32.5 (10.9)	29.3 (8.3)	34.7 (8.0)	31.4 (8.3)	*p =* 0.961	*p* = 0.002

*Mean values (SD) of the systolic and diastolic blood pressure (BP, mmHg), heart rate (bpm), and state anxiety pre and post (90 min) propranolol administration during memory reactivation (i.e., day 2) for both groups. P-values of mixed (time × group) ANOVAs show that all measures significantly decreased over time, an effect that did not differ between the groups*.

### Manipulation check propranolol administration

Systolic BP, diastolic BP and HR significantly decreased 90 min after propranolol intake [main effect of time; *F*_(1, 35)_ = 43.24; *p* < 0.001; η_*p*_^2^ = 0.55; *F*_(1, 35)_ = 8.76; *p* = 0.005; η_*p*_^2^ = 0.20; *F*_(1, 35)_ = 151.76; *p* < 0.001; η_*p*_^2^ = 0.81, respectively]. Since there was no placebo control condition, it is hard to make claims about whether or not propranolol exerted its physiological effect. BP and HR data from day 2, as well as state anxiety scores, are shown in Table 3.

### Online US-expectancy ratings

#### Fear acquisition (day 1)

**Figure 2:**
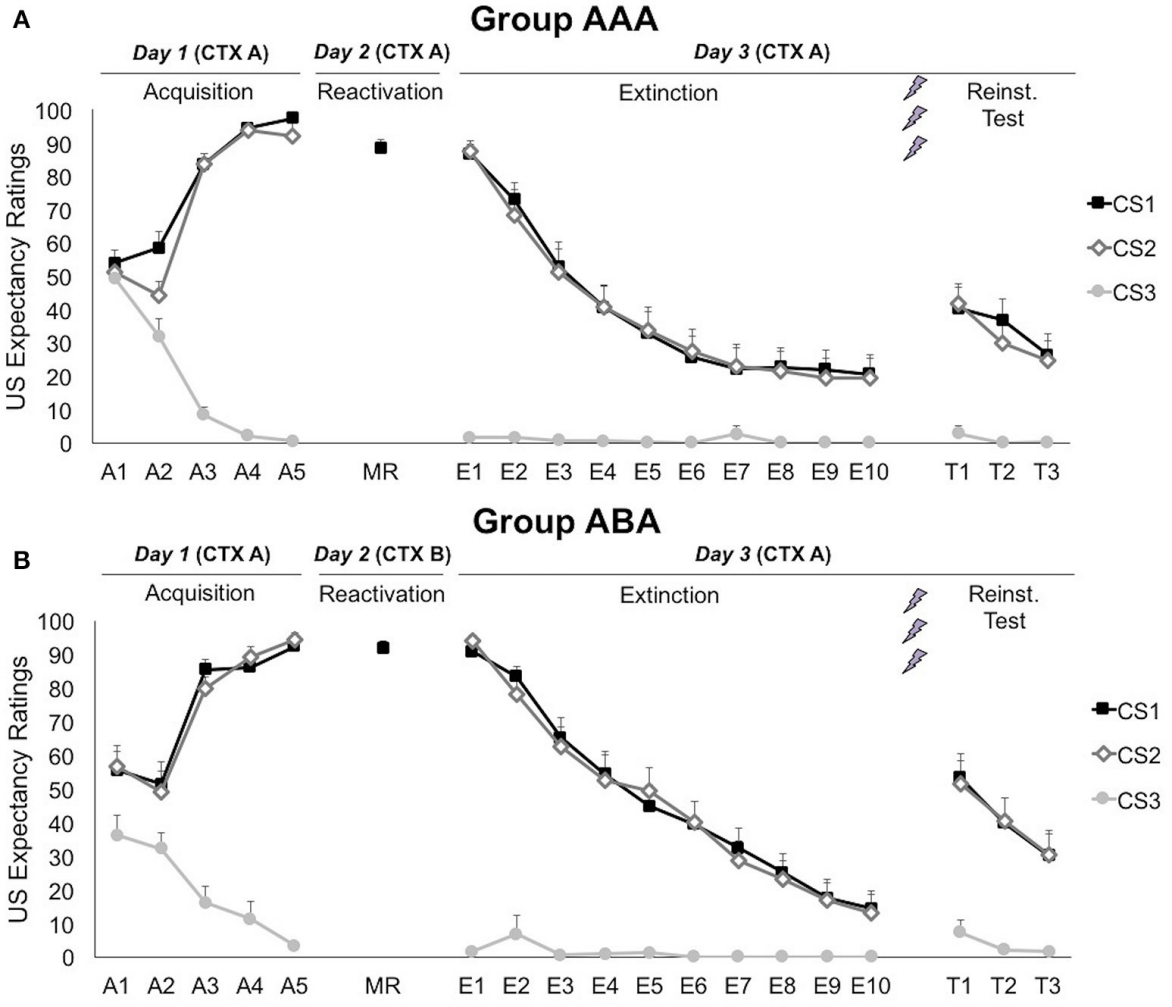
**Mean US expectancy ratings during CS presentation for (A)** reactivation in the training context (CTX A, group AAA, *n* = 20) and **(B)** reactivation in the new context (CTX B, group ABA, *n* = 19). CS1 = reactivated feared stimulus; CS2 = non-reactivated feared stimulus; CS3 = control stimulus. Error bars represent SEM. The labels on the x-axis represent: A, Acquisition; MR, Memory Reactivation; E, Extinction; T, Reinstatement Test.

A 3 × 2 × 2 (stimulus × trial × group) ANOVA showed that differential US-expectancy ratings significantly increased during acquisition, confirming that participants learned to expect the shock after the CSs^+^ and not to expect a shock after the CS^−^ [stimulus × trial; *F*_(2, 66)_ = 140.96; *p* < 0.001; η_*p*_^2^ = 0.81; Figure 2]. This declarative knowledge was acquired similarly in both groups [stimulus × trial × group; *F*_(2, 66)_ = 2.78; *p* = 0.069; η_*p*_^2^ = 0.08].

#### Memory reactivation (day 2)

On day 2, US expectancy during CS1 presentation was not affected by the context [*t*_(37)_ = 1.04; *p* = 0.307], showing that the threat generalized to the new context.

#### Extinction and reinstatement (day 3)

Memory for CS-US contingencies was still intact on day 3, since scores to the CS1 remained significantly higher than to the CS3 [simple contrast; *F*_(1, 33)_ = 2767.38; *p* < 0.001; η_*p*_^2^ = 0.99], but not than the non-reactivated CS2 [simple contrast; *F*_(1, 33)_ = 1.83; *p* = 0.185; η_*p*_^2^ = 0.05]. The significant decrease in differential US-expectancy ratings from the first to the last extinction trial confirms that extinction of US expectancies occurred at the behavioral level [stimulus × trial; simple contrasts CS1 vs. CS3: *F*_(1, 24)_ = 188.24; *p* < 0.001; η_*p*_^2^ = 0.89 and CS2 vs. CS3: *F*_(1, 24)_ = 210.96; *p* < 0.001; η_*p*_^2^ = 0.90]. Nevertheless, US-expectancy for the CSs^+^ did not completely decrease to the level of the CS^−^, but remained significantly higher [main effect of stimulus at E10; simple contrasts CS1 vs. CS3: *F*_(1, 25)_ = 10.60; *p* = 0.003; η_*p*_^2^ = 0.30 and CS2 vs. CS3: *F*_(1, 25)_ = 10.17; *p* = 0.004; η_*p*_^2^ = 0.29]. These effects did not differ between the groups [stimulus × trial × group; *F*_(2, 48)_ = 1.06; *p* = 0.353; η_*p*_^2^ = 0.04].

Delivery of three unexpected USs after extinction resulted in reinstatement of US-expectancy ratings, as shown by an increase in differential ratings from the last extinction trial to the first reinstatement test trial [E10 vs. T1; stimulus × trial; simple contrasts CS1 vs. CS3: *F*_(1, 24)_ = 21.87; *p* < 0.001; η_*p*_^2^ = 0.48 and CS2 vs. CS3: *F*_(1, 24)_ = 32.43; *p* < 0.001; η_*p*_^2^ = 0.58]. This effect did not differ between the groups [stimulus × trial × group; *F*_(2, 48)_ = 0.65; *p* = 0.562; η_*p*_^2^ = 0.03]. Afterwards, expectancy ratings re-extinguished, since differential expectancy decreased from the first to the last test trial [T1 vs. T3; stimulus × trial; *F*_(2, 56)_ = 11.41; *p* < 0.001; η_*p*_^2^ = 0.29]. Expectancy ratings to the CS3 remained stable throughout all phases [A5 vs. E1 vs. T1; main effect of phase; *F*_(2, 62)_ = 2.02; *p* = 0.141; η_*p*_^2^ = 0.06].

### Fear-potentiated startle

#### Habituation

A 10 × 3 × 2 (trial × day × group) ANOVA showed that there was a significant decrease in startle reactivity throughout the 10 habituation trials [main effect of trial; *F*_(6.26, 384.14)_ = 7.37; *p* < 0.001; η_*p*_^2^ = 0.17], which was the same on all days [trial × day; *F*_(10.38, 384.14)_ = 0.81; *p* = 0.628; η_*p*_^2^ = 0.02; Supplementary Material, Figure 2]. However, there was a marginally significant day by group interaction [*F*_(2, 74)_ = 3.08; *p* = 0.053; η_*p*_^2^ = 0.08], indicating that the change in average responding during habituation over days differed between the groups. Since the picture of the context was continuously presented during habituation, differences in average startle responding may reflect differences in contextual fear (Grillon et al., 2004). Follow-up analyses (simple contrasts with day 1 as reference) showed that average startle responding during habitation decreased from day 1 to day 3 in the AAA group [simple effect of day; *F*_(1, 19)_ = 5.06; *p* = 0.036; η_*p*_^2^ = 0.21], while there was a non-significant increase in the ABA group [simple effect of day; *F*_(1, 18)_ = 0.69; *p* = 0.416; η_*p*_^2^ = 0.04]. This group difference is possibly due to the fact that habituation to the training context took place on day 2 in the AAA group, while this was not the case for the ABA group (where reactivation took place in a new context B).

#### Noise alone trials

**Figure 3:**
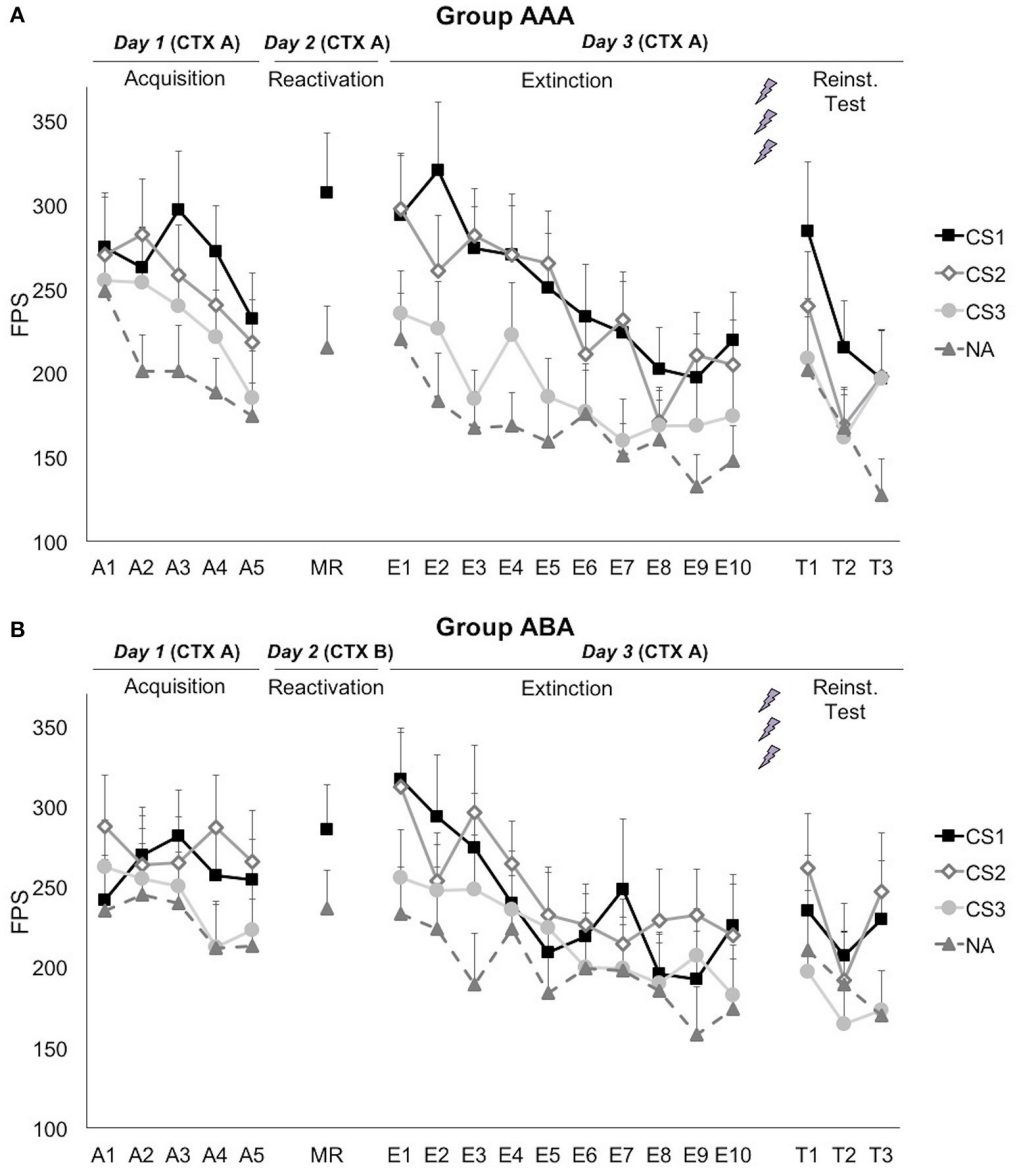
**Mean fear-potentiated startle (FPS) responses show that propranolol HCl did not disrupt fear memory when reactivation occurred in (A)** the training context (group AAA, *n* = 20) or **(B)** in a new context (group ABA, *n* = 19). CS1 = reactivated feared stimulus; CS2 = non-reactivated feared stimulus; CS3 = control stimulus; NA = noise alone trials. The background context (CTX, A or B) was continuously presented. Error bars represent SEM. The labels on the x-axis represent: A, Acquisition; MR, Memory Reactivation; E, Extinction; T, Reinstatement Test.

A series of (trial × group) ANOVAs showed that startle responding to the noise alone (NA) trials significantly decreased during each phase [main effect of trial; Acquisition: *F*_(3.06, 133.16)_ = 3.17; *p* = 0.026; η_*p*_^2^ = 0.08; Extinction: *F*_(6.03, 210.86)_ = 3.91; *p* = 0.001; η_*p*_^2^ = 0.10; Reinstatement Test: *F*_(1.80, 57.67)_ = 4.21; *p* = 0.023; η_*p*_^2^ = 0.12], indicating that habituation to the startle probes still continued during the learning phases (Figure 3). The groups did not differ in startle responding to the NA trials during acquisition [trial × group; *F*_(3.06, 113.16)_ = 1.20; *p* = 0.314; η_*p*_^2^ = 0.03; group; *F*_(1, 37)_ = 0.61; *p* = 0.439; η_*p*_^2^ = 0.02], reactivation [*t*_(37)_ = 0.61; *p* = 0.544], extinction [trial × group; *F*_(6.03, 210.86)_ = 0.47; *p* = 0.834; η_*p*_^2^ = 0.01; group; *F*_(1, 35)_ = 0.43; *p* = 0.517; η_*p*_^2^ = 0.01], or reinstatement test [trial × group; *F*_(1.80, 57.67)_ = 0.36; *p* = 0.697; η_*p*_^2^ = 0.01; group; *F*_(1, 32)_ = 0.46; *p* = 0.504; η_*p*_^2^ = 0.01].

#### Fear acquisition (day 1)

Although, visual inspection of the graphs (Figure 3) suggests (albeit limited) differential fear acquisition, this was not corroborated statistically. A 3 × 2 × 2 (stimulus × trial × group) ANOVA showed that there was no significant increase in differential startle responding from the first to the last acquisition trial [stimulus × trial; *F*_(2, 74)_ = 1.14; *p* = 0.324; η_*p*_^2^ = 0.03; stimulus × trial × group; *F*_(2, 74)_ = 0.16; *p* = 0.857; η_*p*_^2^ < 0.01]. The difference between the three CSs was marginally significant at the end of acquisition [main effect of stimulus at A5; *F*_(2, 74)_ = 3.08; *p* = 0.052; η_*p*_^2^ = 0.08], while there was no baseline difference between the CSs [main effect of stimulus at A1; *F*_(2, 74)_ = 0.83; *p* = 0.441; η_*p*_^2^ = 0.02]. Of importance, when comparing the start of acquisition to the start of extinction training on day 3, a significant stimulus by trial (A1 vs. E1) interaction was found, implying that there was in fact an acquisition effect [*F*_(2, 74)_ = 3.91; *p* = 0.024; η_*p*_^2^ = 0.10].

#### Memory reactivation (day 2)

Fear responding to the reminder trial was significantly higher than the NA trial [main effect of stimulus; *F*_(1, 37)_ = 24.30; *p* < 0.001; η_*p*_^2^ = 0.40]. This difference did not depend on the context during reactivation [stimulus × group; *F*_(1, 37)_ = 2.32; *p* = 0.136; η_*p*_^2^ = 0.06]. Differential fear responding did not significantly change from the last acquisition trial to the reminder trial [stimulus × trial; *F*_(1, 37)_ = 1.04; *p* = 0.314; η_*p*_^2^ = 0.03].

#### Extinction and reinstatement (day 3)

Propranolol after memory reactivation did not attenuate fear memory retention, since differential fear responding (CS1 vs. CS3) did not significantly change from the last acquisition trial (day 1) to the first extinction trial (day 3) [stimulus × trial; *F*_(1, 37)_ = 0.652; *p* = 0.425; η_*p*_^2^ = 0.02]. The absence of a propranolol effect was observed in both groups [stimulus × trial × group; *F*_(2, 74)_ = 0.15; *p* = 0.857; η_*p*_^2^ < 0.01; stimulus × group; *F*_(2, 74)_ = 0.07; *p* = 0.935; η_*p*_^2^ < 0.01], and was regardless of the reactivated stimulus [i.e., whether the spider or the gun picture was used as CS1; stimulus × trial × stimulus category; *F*_(2, 74)_ = 0.06; *p* = 0.939; η_*p*_^2^ < 0.01].

Extinction learning was not successful since there was only a slight, non-significant decrease in differential fear responding [E1 vs. E10; stimulus × trial; *F*_(2, 70)_ = 1.15; *p* = 0.324; η_*p*_^2^ = 0.03], and startle responses to the CSs^+^ remained significantly higher compared to the CS^−^ at the end of extinction [main effect of stimulus at E10; simple contrasts; *F*_(1, 35)_ = 5.64; *p* = 0.023; η_*p*_^2^ = 0.14; *F*_(1, 35)_ = 6.34; *p* = 0.017; η_*p*_^2^ = 0.15, respectively]. However, there was a general decrease in startle responding, indicating habituation to the startle probe [main effect of trial; *F*_(1, 35)_ = 48.47; *p* < 0.001; η_*p*_^2^ = 0.58]. Throughout the extinction phase, differential fear responding was lower in group ABA, compared to group AAA [stimulus × group; *F*_(2, 70)_ = 3.95; *p* = 0.024; η_*p*_^2^ = 0.10]. Visual inspection of the graphs suggests that extinction was actually successful at trial 8, but was then followed by an increase in differential fear responding toward trial 10 (Figure 3). Since differential fear responding was present at the end of the extinction phase, the reminder shocks could not reinstate differential responding [E10 vs. T1; stimulus × trial; *F*_(2, 64)_ = 0.12; *p* = 0.892; η_*p*_^2^ < 0.01]. Subsequent re-extinction learning was not successful [T1 vs. T3; stimulus × trial; *F*_(2, 64)_ = 0.52; *p* = 0.597; η_*p*_^2^ = 0.02].

## Discussion

Administration of propranolol HCl upon memory reactivation did not attenuate fear memory retention on day 3, regardless of whether the memory was reactivated in the training context or in a novel context. At the start of the retention test on day 3, fear responding to the reactivated fear-conditioned stimulus (CS1) was equal to the non-reactivated fear-conditioned stimulus (CS2), and significantly higher than to the control stimulus (CS3). These results suggest that memory destabilization was not triggered in either context. It is import to emphasize that the basic features of associative fear learning (i.e., fear acquisition, extinction) were established only weakly in the current study, implying that the results should be interpreted with caution. We will get back to this issue later on in the discussion. Nevertheless, the current findings are in contrast with previous studies from our lab, which clearly demonstrated that propranolol administration after memory reactivation can attenuate the emotional expression of fear memory while leaving declarative memory unaffected (Kindt et al., 2009; Soeter and Kindt, 2010, 2011, 2012a,b, 2013, 2015a,b; Sevenster et al., 2012, 2013, 2014). In those experiments it was repeatedly shown that propranolol administration 90 min before or directly after memory reactivation can block the retention and return of fear memory, as evidenced by disrupted fear responding that persisted after reinstatement and at 1 month follow-up (Soeter and Kindt, 2010). The propranolol effect was not observed when propranolol was administered in the absence of cue-elicited memory reactivation (Kindt et al., 2009).

In agreement with the current results, the disruptive effect of propranolol could not be replicated in a series of preliminary pilot studies conducted by the same experimenter using different fear acquisition parameters (*N* = 51, data not shown). In addition, we had one study (among at least 11 successful studies) in which we could also not observe fear reduction by either extinction learning or the induction of post-reactivation amnesia (Bos et al., 2014). It should be noted that also in the present study, lack of evidence for reconsolidation or its interference was accompanied by non-significant regular extinction learning. There is one additional report in the literature of a failure to replicate our previous propranolol findings, by an independent group, despite the use of a similar protocol in healthy subjects (Thome et al., 2016). In line with these inconsistent findings, several studies were not able to observe the disruption of memory reconsolidation by a behavioral intervention (i.e., retrieval-extinction procedure), which was initially documented by Monfils et al. (2009) in rats, and by Schiller et al. (2010) in humans (Fone and Porkess, 2008; Pérez-Cuesta and Maldonado, 2009; Chan et al., 2010; Ma et al., 2012; Soeter and Kindt, 2012b; Auber et al., 2013; Kindt and Soeter, 2013). These difficulties with the (conceptual) replication of pharmacological and behavioral memory interference illustrate that the conditions to trigger memory reconsolidation depend on subtle manipulations (Sevenster et al., 2014; Kindt and van Emmerik, 2016), which may point to restrictions for a swift translation to clinical studies.

Several explanations for these remarkable discrepancies have been formulated in the literature. First, the failed replications have been attributed to methodological differences between studies with contrasting results, such as the amount of CS-US parings, CS type, reinforcement scheme, US characteristics, drug dose, or reactivation parameters (Auber et al., 2013; Meir Drexler and Wolf, 2016; Thome et al., 2016). Meta-analyses have indeed identified several specific methodological variables that may moderate the success of reconsolidation interference (Das et al., 2013; Lonergan et al., 2013; Kredlow et al., 2016). However, given that the evidence for those mediators is correlational, they should be regarded as speculative in the absence of direct experimental evaluation. Second, the absence of amnestic effects has often been attributed to the presence of so-called “boundary conditions.” These conditions refer to certain memory characteristics such as memory strength, age, and type (e.g., inhibitory avoidance) that may render memories less sensitive to amnestic interventions (Suzuki, 2004; Wang et al., 2009; Muravieva and Alberini, 2010; Robinson and Franklin, 2010), or to certain participant characteristics such as sex (Meir Drexler and Wolf, 2016) or high trait anxiety (Soeter and Kindt, 2013). However, given the highly similar methodology of the current study compared to those of previous successful reports in our lab, it seems unlikely that any of these conditions would apply to this experiment. More specifically, methodological similarities include, among others, the use of IAPS pictures as conditioned stimuli (i.e., cup, spider, and gun each presented five times), an electrical shock as unconditioned stimulus, the reinforcement schedule (i.e., 80%), instructions for the participants, apparatus, data processing methods, memory age (i.e., 24 h), dose of propranolol (i.e., 40 mg), and parameters of reactivation, extinction, and reinstatement (Soeter and Kindt, 2011, 2012b), with the exception of the background pictures as meaningful contexts of the CSs in the current study. Participant characteristics can also not account for our discrepant results, since average trait anxiety, anxiety sensitivity, spider fear, age, and male-female ratio are within the range of those in previous studies from our lab. Thus, purported boundary conditions or moderators cannot readily account for the current failure to replicate the fear-reducing effect of propranolol administration. Therefore, our findings remain unclear and we can merely speculate about possible explanations in the following paragraphs.

First, despite the fact that differential fear responding on the first trial on day 3 reached a large effect size, it should be noted that differential fear responding at the end of day 1 was considerably smaller than in previous studies using a highly similar acquisition procedure (η_*p*_^2^ = 0.03 vs. η_*p*_^2^ ≥ 0.33; Soeter and Kindt, 2011, 2012b). This discrepancy either indicates that learning was less effective in the current experiment, or it reflects a nosier measure of fear-potentiated startle (FPS) than in our previous studies. Based on the first possibility (i.e., relatively weak initial fear acquisition), one might argue that fear learning was insufficiently strong to induce a memory trace that is sensitive to destabilization. Therefore, we created a subsample of participants with successful fear acquisition (i.e., average startle responding during the last two acquisition trials is higher for the CSs^+^ than the CS^−^), including 11 participants (5 of group AAA). Fear acquisition was strong in this subsample (i.e., stimulus × trial interaction, η_*p*_^2^ = 0.51). Although, this small sample size does not allow statistical testing for a propranolol effect, visual inspection of the differential startle responses on day 3 suggests the same pattern as in the overall sample (i.e., no effect of propranolol and no successful extinction learning in either group, data not shown). Thus, in a subsample of participants with strong levels of fear acquisition, reconsolidation and extinction did not seem to have been induced either. This suggests that the weak fear acquisition on day 1 in the current study does fully not account for propranolol's failure to interfere with reconsolidation.

Second, similar to the weak fear acquisition on day 1, extinction of the emotional fear response was weak-to-absent in the current sample. In addition, fear responding generalized to the control stimulus, as shown by a differential (CS3 vs. NA) increase in fear responding from day 1 to day 3. These unexpected observations may again imply a nosier measure of FPS responses or they may indicate that the participants failed to rely on the CS-US -and CS-no US contingencies at the emotional level (albeit they did learn these contingencies on the declarative level). Of note, the papers by Bos et al. (2014) and Thome et al. (2016), in which propranolol failed to affect fear memory, report similar observations of weaker fear acquisition and no or minimal extinction learning than in most of our previous studies, and generalization of the conditioned fear responding toward the control stimulus. These general learning deficits may possibly explain why our results run counter to the bulk of previous studies from our lab. Although, US expectancy ratings indicate that the participants successfully learned the CS-(no) US contingencies, this does not necessarily imply associative learning at the emotional level (i.e., no significant changes in differential FPS responding during conditioning and extinction training). If the participants' emotional learning did not rely on the CS-(no)US contingencies, the CS-no US presentation during reactivation might have failed to induce a sufficiently clear prediction error (PE, mismatch between actual and expected outcomes). As a result, memory may not have been destabilized and there was no opportunity for propranolol to exert its fear-reducing effect. However, it should be noted that this argument, stating that the failure of propranolol possibly implies the absence of PE, is somehow circular, as it is based on previous evidence indicating that PE is required for memory destabilization and, in turn, for propranolol to interfere with the memory trace (Sevenster et al., 2013; Exton-McGuinness et al., 2015; Fernández et al., 2016). In addition, this explanation is purely hypothetical, since there was no independent measure of PE in this experiment (see Limitations).

We hypothesized that memory destabilization would be induced upon presentation of the reactivation cue in the original learning context (i.e., in group AAA) but not in a novel context (i.e., in group ABA), since a context change would induce too much novelty for memory updating to occur. However, given that fear memory did not appear to be destabilized under the usual conditions (i.e., AAA) in the present study, contextual novelty might have actually boosted memory destabilization, as it has previously been shown that new contextual information can promote the destabilization of otherwise insusceptible fear memories (Winters et al., 2009; Jarome et al., 2015). These two animal studies showed that administration of a protein-synthesis blocker after a reactivation session disrupted the memory trace only when novel contextual information was presented, suggesting that new contextual information induced reconsolidation of the previously-encoded context representation (Finnie and Nader, 2012). Following this line of reasoning, the presentation of novel contextual information in our paradigm might have actually boosted reconsolidation-dependent updating of (a part of) the memory that could not be updated in the training context. However, our results show that cued fear retention was not affected by contextual novelty either. Nevertheless, merely the context-dependent portion of the memory could have been updated, which would result in a propranolol-induced decrease in contextual fear. Since there was no indication of contextual fear learning, our paradigm does not allow assessment of contextual fear memory updating (Grillon et al., 2004). In any case, it can be concluded that there is no evidence that new contextual information blocked or allowed destabilization of cued fear memory in the current experiment.

Finally, some limitations of the current study need to be acknowledged. First, as previously mentioned, there was only moderate fear acquisition on day 1 in the overall sample. This emotional learning deficit can be due to the use of a background context, which was common to all CSs. The context could have distracted the participant's attention toward the CSs and therefore possibly hindered differential fear acquisition. In addition, two (rather than one) CSs^+^ were used, which complicates fear learning and necessitates the use of eight (instead of four) US exposures in our current study, thereby increasing the risk of US habituation. Although, there was no indication of habituation in previous studies using two CSs^+^ by Soeter and Kindt (2011, 2012b), several observations in our experiment indeed support the idea of US habituation. For example, since US intensity was relatively high compared to previous experiments, participants already received a higher amount of shocks during the work-up procedure before actual fear conditioning. This increased exposure may have facilitated habituation to the sensation of receiving a shock. Indeed, the average decrease in subjective US unpleasantness from day 1 to day 3 suggests that some habituation to the shock took place. This decrease was not observed in a previous study in which we assessed PE (Sevenster et al., 2013). Remarkably, participants also rated the startle probe as more unpleasant than the shocks (Table 2), indicating that the shock might have been too weak to induce anticipatory fear. Of note, a recent meta-analysis of reactivation-extinction studies suggests that the effect of conducting extinction during reconsolidation in preventing fear recovery in humans is more reliable in between-subjects designs than in within-subject designs (Kredlow et al., 2016). At any rate, the relatively weak acquisition effect and the lack of clear extinction to the control CS^+^ (CS2) may well bear a critical relation to our failure to observe amnestic effects of propranolol administration.

Second, it remains debatable whether a picture of a room effectively constitutes a context. Nevertheless, several studies have successfully demonstrated context effects on memory and/or fear learning (e.g., fear renewal) by using background pictures or even colors (Dibbets et al., 2008; Neumann and Kitlertsirivatana, 2010; Soeter and Kindt, 2012a; Mertens and De Houwer, 2015). Although, it would have been better (but not feasible) to actually use different rooms instead of pictures of rooms on a computer screen, the use of these background pictures seems to be a valid approach to investigate context effects on fear learning and memory.

Third, our current design did not allow us to independently measure the presence of PE during reactivation. Sevenster et al. (2013, 2014) found that reactivation-induced changes in US expectancy could indicate that PE occurred. This behavioral index of PE could show whether memory was destabilized, independent of the outcome of the amnestic intervention. It bears mentioning however that the experimental protocol in these studies differed from the protocol used in the current study. Other previous experiments, using a partially-reinforced learning session without instructions on the contingencies, produced some uncertainty at the end of learning. These studies illustrate that a decrease in US expectancies after a non-reinforced reactivation session was not necessary for destabilization, since an amnestic effect was observed in the absence of such a change in US expectancies (Soeter and Kindt, 2011, 2012b, 2015b). It is thus not straightforward to interpret the meaningfulness of changes in threat expectancy in this study (i.e., with an 80% reinforcement schedule and no explicit instructions about CS-US contingencies).

## Conclusion

The induction of post-reactivation amnesia offers a promising therapeutic avenue to persistently reduce the strength of maladaptive memories. Although many studies report robust and large amnestic effects, several others fail to replicate these findings. These discrepant results indicate that the requirements for triggering memory destabilization can be difficult to fulfill, and in addition, they largely remain unclear. The current study used a highly similar design as previous studies that convincingly showed evidence for memory reconsolidation interference. Hence, our failure to replicate memory reconsolidation interference cannot simply be explained by methodological differences or alleged boundary conditions on memory destabilization. More research is required to clarify the precise conditions under which memories can be destabilized. Future studies might use designs in which prediction error can be assessed independently from the mnemonic outcome. Meanwhile, the present results emphasize that the success of human fear conditioning studies (including fear acquisition, extinction, and reconsolidation) depends on (unknown) subtle differences in the experimental protocols and procedures.

## Ethics statement

This study was carried out in accordance with the recommendations of the local ethics committee at the University of Amsterdam with written informed consent from all subjects.

## Author contributions

MK and NS contributed to the design of the work. NS was responsible for acquisition and analysis of the data. MK, NS, and TB contributed to the interpretation of the results. NS drafted the article, while MK and TB critically revised the article.

## Funding

Preparation of the paper was funded by the ERC Consolidator Grant 648176 awarded to TB.

### Conflict of interest statement

The authors declare that the research was conducted in the absence of any commercial or financial relationships that could be construed as a potential conflict of interest.
