# Supplementary material for: In Search for Boundary Conditions of Reconsolidation: A Failure of Fear Memory Interference
Source: Front Behav Neurosci. 2017 Apr 19;11:65. doi: 10.3389/fnbeh.2017.00065 (PMC5395559; doi:10.3389/fnbeh.2017.00065)
Supplement: Supplementary file 1 [file DataSheet1.pdf]

Supplementary Material

In Search for Boundary Conditions of Reconsolidation: A Failure of Fear Memory Interference

Natalie Schroyens, Tom Beckers, Merel Kindt*

*** Correspondence:** Merel Kindt: M.Kindt@uva.nl

Supplementary Figures

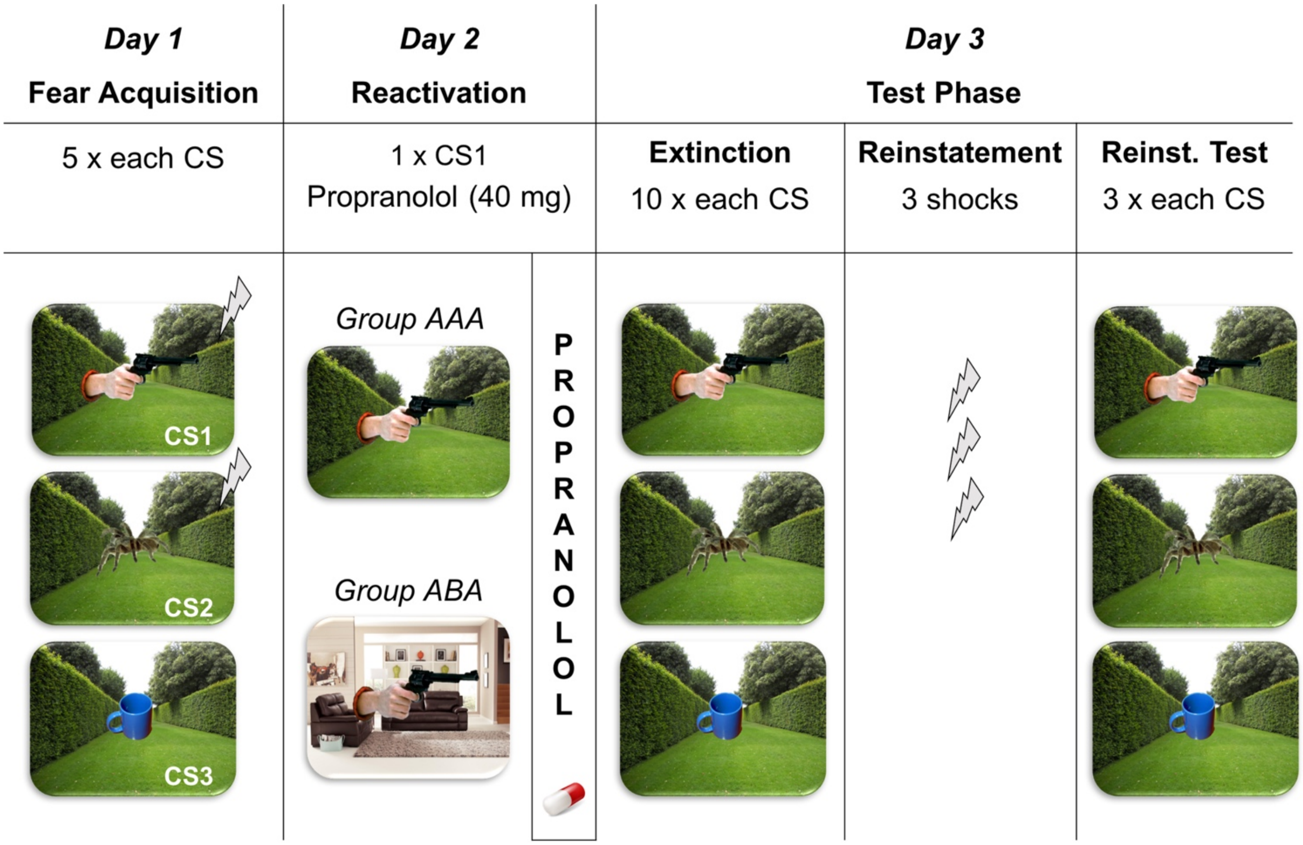

Supplementary Figure 1. A more detailed overview of the experimental protocol. CS1 = reactivated conditioned stimulus (i.e., gun), CS2 = non-reactivated conditioned stimulus (i.e., spider), CS3 = control stimulus (i.e., cup). Background pictures of a garden or a living room are used to represent contexts. The use of the pictures as CS1 and acquisition context were counterbalanced.

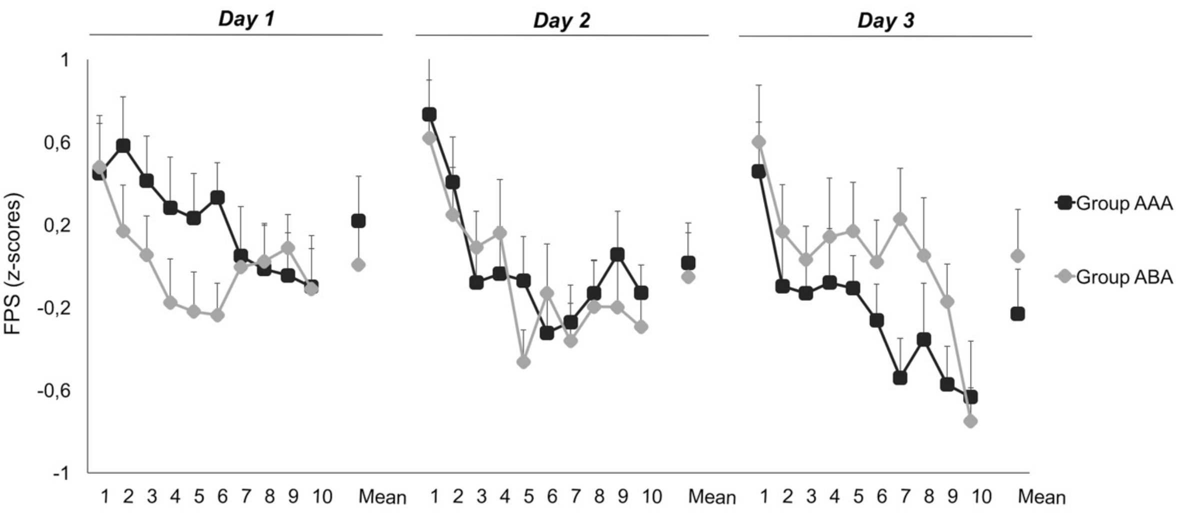

Supplementary Figure 2. Average startle responding during the habituation trials (1-10) before acquisition (day 1), reactivation (day 2) and extinction (day 3) (*N* = 39). The mean startle response during habituation per day is also shown. The context picture (i.e., training context (A) or new context (B)) was continuously presented throughout habituation. Error bars represent SEM. *Z*-scores were calculated based on all habituation trials within one subject.
